# Nonunion of the so-called acromion: a systematic review with consideration of the terminology

**DOI:** 10.1007/s00402-023-04912-z

**Published:** 2023-06-14

**Authors:** Kiyohisa Ogawa, Noboru Matsumura, Atsushi Yoshida, Wataru Inokuchi

**Affiliations:** 1 Department of Orthopedic Surgery, Eiju General Hospital, 2-23-16 Higashi-Ueno, Taito-ku, Tokyo, 110-8645 Japan; 2 Department of Orthopedic Surgery, School of Medicine, Keio University, 35 Shinanomachi, Shinjuku-ku, Tokyo, 160-8582 Japan; 3 Department of Orthopedic Surgery, National Hospital Organization Saitama Hospital, 2-1 Suwa, Wako City, Saitama 351-0102 Japan; 4 Department of Orthopedic Surgery, Eiju General Hospital, 2-23-16 Higashi-Ueno, Taito-ku, Tokyo 110-8645 Japan

**Keywords:** Scapular fracture, Acromion fracture, Scapular spine fracture, Nonunion, Terminology

## Abstract

**Introduction:**

There is no widely accepted standard for the classification and treatment of traumatic acromion/scapular spine fracture nonunion due to the scarcity of this condition and the confusion of terminology.

**Materials and methods:**

PubMed and Scopus were searched using “scapular fracture” and “acromion fracture” or “scapular spine fracture” as search terms. The inclusion criteria were English full-text articles concerning acromion/scapular spine fracture nonunion that described patient characteristics and presented appropriate images. The exclusion criteria were cases without appropriate images. Citation tracking was conducted to find additional articles and notable full-text articles written in other languages. Fractures were classified using our newly proposed classification system.

**Results:**

Twenty-nine patients (19 men, 10 women) with 29 nonunions were identified. There were four type I, 15 type II, and 10 type III fracture nonunions. Only 11 fractures were isolated. The mean period from initial injury to final diagnosis was 35.2 ± 73.2 months (range 3–360 months) (*n* = 25). The most frequent cause of delayed diagnosis was conservative treatment for fracture in 11 patients, followed by oversight by the physician in 8. The most common reason for seeking medical advice was shoulder pain. Six patients received conservative therapy, and 23 received operative treatment. Fixation materials included various plates in 15 patients, and tension band wiring in 5. Bone grafting was performed in 16 patients (73%, 16/22). Of the 19 surgically treated patients with adequate follow-up, the outcome was rated excellent in 79%.

**Conclusions:**

Isolated acromion/scapular spine fracture nonunion is rare. Fracture type II and III, arising in the anatomical scapular spine, accounted for 86% of the fractures. Computed tomography is required to prevent fracture oversight. Surgical therapy produces good stable results. However, it is important to select the appropriate surgical fixation method and material after considering the anatomical characteristics of the fracture and stress on the fractured portion.

**Level of evidence:**

V

## Introduction

The shoulder is a complex joint that ranges from the sternoclavicular joint to the glenohumeral joint and consists of multiple anatomical joints and joint-like structures as well as three bones (clavicle, scapula, and humerus). In this complex joint, the acromion plays an important role as (1) a connecting part with the clavicle via the acromioclavicular joint, (2) an attachment site of the acromioclavicular ligaments and coracoacromial ligament, (3) a bony part of the coracoacromial arch contributing to superior stability of the glenohumeral joint, (4) and the origin or insertion sites of two major muscles (the trapezius and deltoid). Consequently, fractures of the acromion have many effects on the function of the shoulder joint and neighboring musculoskeletal systems, even though acromion is a small bone projection.

Scapular fractures account for approximately 1% of all fractures and 3%–5% of fractures of the shoulder girdle [1–4]. Therefore, scapula fractures have traditionally been considered relatively rare fractures, but the number of diagnosed scapular fractures has increased recently due to the increasing use of chest computed tomography (CT) for trauma patients [5]. Acromion fracture accounts for 8–18% of scapular fractures in studies using plain radiography [2, 4, 6–12]. Combining acromion fractures and scapular spine fractures range from 9 to 29% [2, 7, 13–15]. However, while the acromion is a relatively small and irregularly quadrangular bone in anatomical terms, the term ‘acromion’ is used surgically and clinically to indicate a much larger region [16]. There is an inconsistency in the nomenclature for acromion fracture in the literature [17–19]. Some authors consider only the area of the anterior bony protrusion from the lateral scapular spine as the acromion, while the rest is referred to as the lateral extension of the scapular spine, or more simply as the scapular spine [14, 15, 20–33]. However, other authors consider the acromion to be the entire bony prominence lateral to the spinoglenoidal notch [7, 9, 34–41]. Consequently, fractures at the same anatomical location are clinically referred to by many different names; for example, the sagittal fracture from the superior crest of the scapular spine to the spinoglenoidal notch is referred as a fracture of the acromion [42], fracture of the base or neck of the acromion or scapular spine [43–46], and scapular spine fracture at the base of the acromion [47, 48]. These differences have given rise to misleading statements in the literature.

The purpose of the present review was to systematically evaluate the available literature to clarify the current concept of nonunion after traumatic acromion and scapular spine (acromion/spine) fractures, which have conventionally not been evaluated due to the rarity of these fractures. We briefly introduce the anatomical definition and developmental processes of the acromion/spine, as knowledge of these topics is needed to understand acromion/spine fractures and resolve inconsistencies in the nomenclature used in the literature. In accordance with anatomical and developmental consideration, we then proposed a new classification system for traumatic fractures arising lateral to the spinoglenoidal notch.

### What is the acromion?

#### Anatomical definition

The most authoritative anatomical texts are consistent in their definition of the acromion: *The acromion projects forwards, almost at right angles, from the lateral end of the spine, with which it is continuous. The lower border of the crest of the spine becomes continuous with the lateral border of the acromion at the acromial angle. The medial border of the acromion is short* [29, 32].

### Consideration from the developmental process

**Fig. 1:**
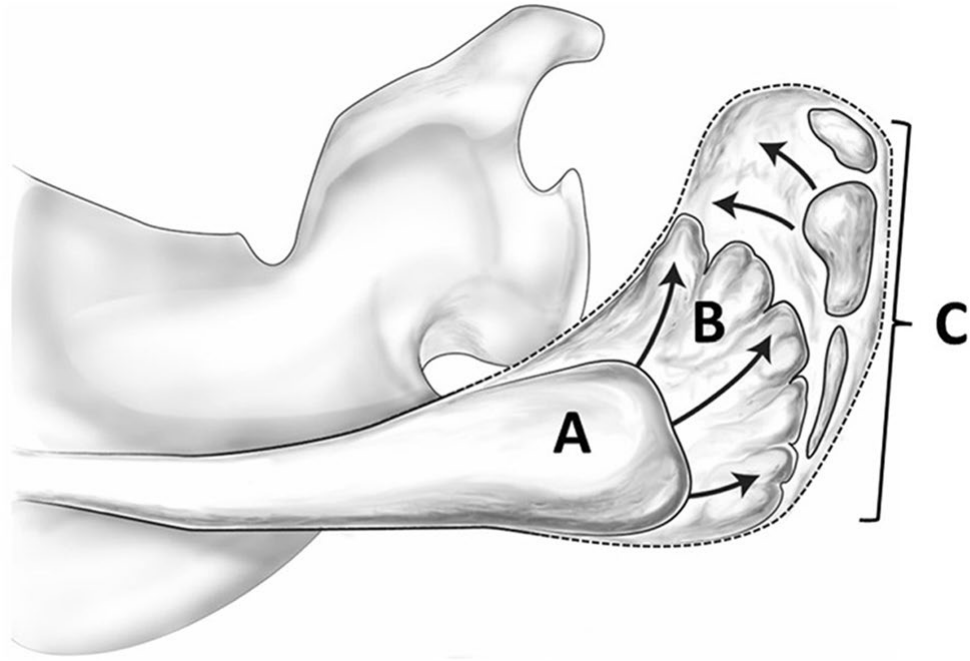
Developmental process of the acromion. At birth, the ossified spinous process ends in a bulbous lateral extension with an epiphyseal surface (**A**). The base of the acromion is formed by an extension from the scapular spine (**B**). Multiple secondary ossification centers for the acromion arise between 14 and 16 years and gather into three centers along the lateral acromial area, namely the preacromion, mesoacromion, and metacromion from the anterior tip of the acromion (**C**)

In the cartilaginous scapula, the primary ossification center for the body appears around 7 to 8 fetal weeks [36, 49]. Ossification expands endochondrally and intramenbranously and reaches the level of the base of the scapular spine [27, 36]. At birth, the ossified spinous process ends in a bulbous lateral extension, which bears an epiphyseal surface [27, 36]. The base of the acromion is formed by an extension from the scapular spine, and generally extends from just medial to the acromial angle and advances anteriorly toward the acromioclavicular joint with growth. Multiple secondary ossification centers for the acromion arise between 14 and 16 years of age [27], although there is a considerable amount of variation in the time of appearance and number of ossification center [50–53]. These secondary ossification centers gather into three centers along the lateral edge (preacromion, mesoacromion, and metacromion) from the anterior tip of the acromion [20, 27, 36, 49, 54]. The last ossification center reaches the acromial angle. These findings were also recently confirmed in detail in vivo by magnetic resonance imaging (MRI) [52, 54, 55] (Fig. 1). Complete fusion between the base of the acromion and acromial epiphysis does not tend to occur before 20 years of age, with the most concentrated period of activity being between 18 and 20 years [27]. Some researchers have demonstrated that socioeconomic status plays a much greater role in bone maturation speed compared with ethnicity with a poor socioeconomic status slowed bone maturation [53, 56, 57].

### Proposed classification

**Fig. 2:**
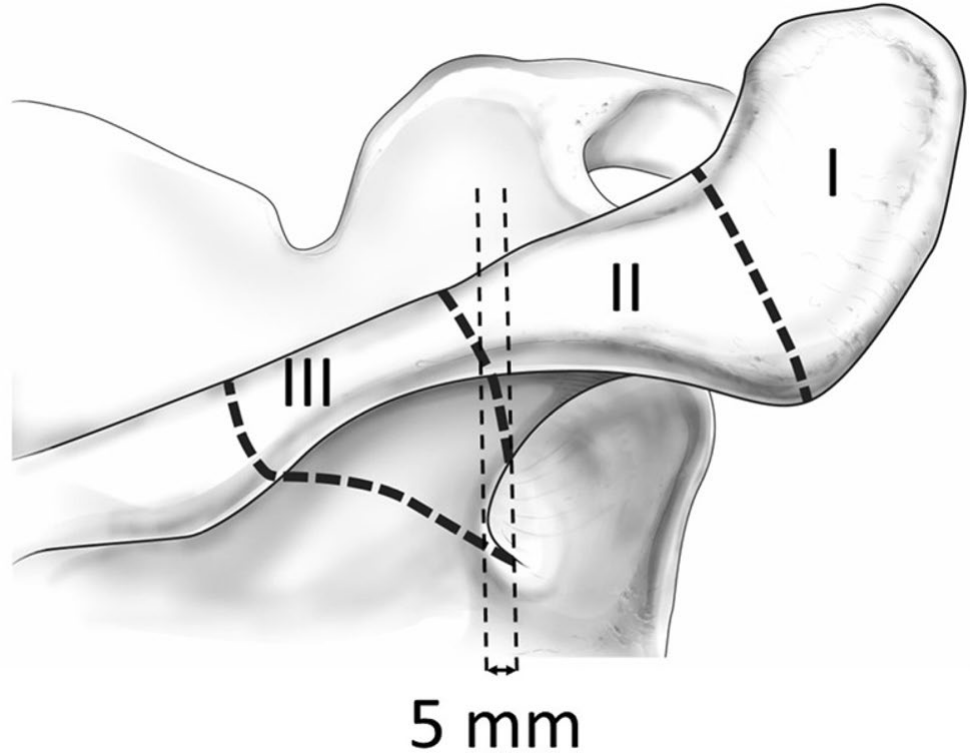
Proposed simple classification system using the acromion angle and spinoglenoidal notch as border points. Type I: A fracture of the lateral end being from the anterior margin of the acromion to the acromion angle. Type II: A fracture of the posterior edge being from the acromion angle to 5 mm lateral to spinoglenoidal notch. Type III: A fracture that extends from the crest of the scapular spine to the spinoglenoidal notch or 5 mm lateral to this notch.

Terminological inconsistency in clinical and anatomical fields hinders understanding of the various conditions that occur in the region from the scapular spine to the acromion region. We consider that the classification system of fractures in this region based on anatomical and developmental aspects is a useful means of resolving terminological confusion and leading to better understanding. Therefore, we proposed a new simple classification system using the acromion angle and spinoglenoidal notch as border points that can be easily recognized clinically and by imaging, and used this classification system in the present review (Fig. 2).

Type I: A fracture of the lateral end existing from the anterior margin of the acromion to the acromion angle.

Type II: A fracture of the posterior edge being from the acromion angle to 5 mm lateral to the spinoglenoidal notch.

Type III: A fracture that extends from the crest of the scapular spine to the spinoglenoidal notch or 5 mm lateral to this notch.

Fractures in which the posterolateral end of the fracture line is exactly at the acromion angle are considered type I fracture because the metacromion forms part of the acromion angle [20, 52, 54, 55]. Type III fracture is characterized by a wide fracture surface, and this characteristic is prominent up to 5 mm lateral to the spinoglenoidal notch. However, further lateral fractures lead to rapid reduction of the fracture surface and loss of type III features. Fractures of the scapular spine medial to the spinoglenoidal notch, in which the lateral fragment does not separate from the scapular body, are not included in this classification system, because the clinical presentation and disability are completely different from type I–III fractures in which the lateral bony fragments are completely disconnected from the scapular body.

## Materials and methods

**Fig. 3:**
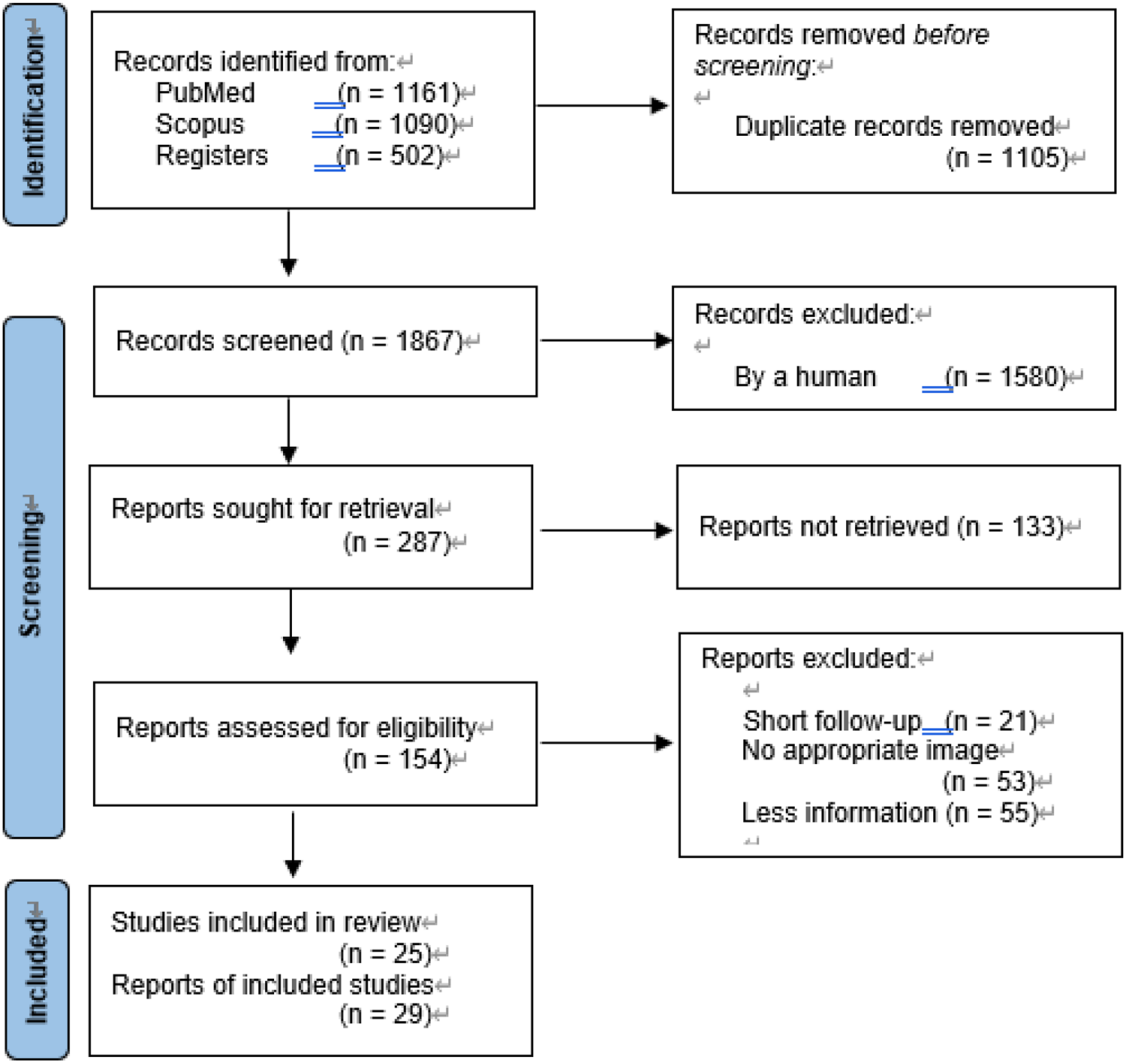
PRISMA flowchart of the study

This systematic review was conducted in accordance with the Preferred Reporting Items for Systematic Reviews and Meta-Analyses Protocols guidelines [58]. The literature search was performed from July 2022 to September 2022, and the publication years of the included articles ranged from 1900 to 2021. The PubMed and Scopus databases were searched using the terms “scapular fracture” and “acromion fracture” or “scapular spine fracture” to identify relevant studies. Two reviewers (K.O., N.M.) independently conducted the literature search and review. The inclusion criteria were English full-text articles concerning acromion and/or scapular spine nonunion after an acute traumatic fracture that described the patients’ characteristics and presented the appropriate images (radiography, CT, or MRI) to confirm the details of the fracture line or classified the fracture using a classification system that indicates the anatomical fracture location [26]. The exclusion criteria were descriptive articles or cases, articles or cases without appropriate images to enable the evaluation of the injury details, stress fractures, a young patient with an unfused acromion physis, and fractures in patients with a history of surgical intervention that weakens acromion/spine. Citation tracking was conducted to find additional related English articles and notable full-text articles written in other languages, which were carefully selected and added to the qualitative synthesis (five studies) [22, 44, 59–61] (Fig. 3).

After the title and abstract assessment, 287 full-text articles were further evaluated. From these studies, the cases of nonunion of the acromion or scapular spine and cases judged as nonunion of the acromion or scapular spine from the provided images and description were selected. Nonunion was defined as a fracture that had not united or was lacking an appropriate bone reaction at the fracture site more than 3 months after the injury or surgery [37]. A total of 129 studies were excluded because the period from the accident/surgery was less than 3 months or there were insufficient data. Consequently, 25 studies were finally included in the analysis. Each patient was reviewed regarding age, sex, cause of injury, fracture type, concurrent injuries, cause of chronicity, type of treatment, and outcome. The fractures were categorized using the abovementioned novel classification system.

The rate of concurrent shoulder girdle injuries, fixation method used for each fracture type, an excellent rate for the outcome of each fracture type in surgically treated cases, and rate of reported isolated acromion/spine fracture were evaluated using the Chi-squared test with the significance level set at *p* < 0.05.

## Results

### Patient characteristics

There were 29 patients with 29 nonunion of acromion/spine fractures described in 25 articles. The patients comprised 19 men and 10 women with a mean age at injury or diagnosis of 44.1 ± 16.2 years (range 16 to 75 years). The injured side was right in 15 patients, left in 11, and unknown in three. Of the eight patients whose dominant side was specified, three patients were injured on the dominant side and five were injured on the non-dominant side. The causes of initial trauma were various traffic accidents/crashes in 13 patients, a fall or fall from a height in five, injuries sustained during or due to sports activities such as skiing and gymnastics in two (including an apparent complete fracture as the final stage of stress fracture), iatrogenic accidents during surgical procedures in two, excessive muscle force in one, other accidents in three, and uncertain or unknown in three. There were two patients with iatrogenic acromion/spine fracture; one was a 34-year-old woman with psychological problems who had a fracture confirmed 2 months after open anterior acromioplasty for chronic subacromial impingement and was treated conservatively [45], while the other was a 67-year-old man whose fracture occurred during arthroscopic subacromial decompression for a massive rotator cuff tear [61] (Table 1 [62–66]).

**Table 1:** Patient characteristics in published cases of fracture nonunion of the acromion/scapular spine

Case no	Report year	Author	Age (years)/Sex/Side	Cause	Fx. type	Complications	SSSC injury	Period from injury to final Tx. & cause of delay
1	1942	Horn [59]	49/M/R	Quetionable	II	Coracoid fx	Double	Acute
2	1970	Gordes and Hessert [22]	22/M/R	Traffic accident	II	Scapular neck fx	Double	6 M Overlooked
3	1970	Gordes and Hessert [22]	59/F/R	Trapped in closing tram door	III	Scapular neck fx	Double	14 M Left untreated & overlooked
4	1983	Mick and Weiland [62]	57/M/R	Fall during syncopal episode	II			5.5 M (1.5 M Left untreated & 4 M Cons.)
5	1987	Morisawa et al. [44]	40/M/R	Traffic accident	III	Cranial fx., multiple rib fx., hemothorax		3 M Overlooked
6	1992	Marr and Misamore [45]	34/F/R	Anterior acromioplasty	III			
7	1993	Robinson and Court-Brown [63]	59/M/L	Direct blow	III			3.5 M Cons
8	1994	Kuhn et al. [37]	30/F/L	Lifting a heavy patient	I			10 M Cons
9	1994	Warner & Port [64]	16/F/L	Gymnastics	II			8 M Cons
10	1995	Naested et al. [75]	25/F/R	Trauma during alpine skiing	I			24 M Cons. & Overlooked
11	1998	Böhm [72]	40/M/R	Fall from a height	III			360 M Left untreated
12	1999	Dounchis et al. [76]	23/M/R	Traffic accident	II	Multiple rib fx., pneumothorax, fx.-disl. of T8		14 M Cons
13	2003	Charlton et al. [47]	25/M/R	Traffic accident	III	Multiple rib fx., pneumothorax, scapular body + coracoid + LT fx., suprascapular nerve palsy	Triple	9 M Cons
14	2006	Lumbreras et al. [60]	59/F/R	Traffic accident	III	Solid viscera inj., tibia fx., ACL rupture		60 M Overlooked even after ORIF
15	2008	As-Sultany et al. [48]	39/M/L	Fall	III			6 M Cons
16	2008	Richards and Curtis [68]	43/M/L	Traffic accident	I	Posterior ACJ disl	Double	5 M Left untreated
17	2009	Anavian et al. [40]	43/F/L	Explosion	II	ACJ disruption, clavicular fx	Double	4 M Cons
18	2011	Liodakis et al. [61]	67/M/R	ASD intraoperative	I			60 M Overlooked
19	2011	Moallemzadeh & Gosens [71]	40/F/L	Traffic accident	II	Bilateral patellar fx		72 M Overlooked & Cons
20	2014	Copuroglu et al. [65]	50/M/L	Traffic accident	III			24 M Overlooked
21	2017	Muiño et al. [66]	36/M/L	Traffic accident	II	Multiple rib fx		24 M Cons
22	2018	Tladi [74]	35/M/R	Traffic accident	II	Contralateral femoral fx		24 M Overlooked
23	2019	Hess et al. [70]	75/M/L	?	II	Serial rib fx., pneumothorax		6 M Cons
24	2019	Hess et al. [70]	72/M/R	?	II	Vertebral body fx. (Th6)		Acute
25	2019	Almustafa [73]	36/M/R	Traffic accident	II	Contralateral distal radius fx		9 M Cons
26	2020	Konstantinidis et al. [69]	41/M/L	Traffic accident	II			Acute
27	2021	Ogunleye et al. [67]	56/F/	Fall from a height	II	ACJ dislocation, coracoid fx	Triple	≥ 5 M
28	2021	Ogunleye et al. [67]	34/M/	Gunshot	III	ACJ dislocation, coracoid fx	Triple	120 M
29	2021	Ogunleye et al. [67]	74/F/	Fall down stairs	II	Coracoid fx	Double	≥ 5 M

*ASD* arthroscopic subacromial decompression, *ACJ* acromioclavicular joint, *ACL* anterior cruciate ligament, *SSSC* superior shoulder suspensory complex,

*M* male, *F* female, *R* right, *L* left,  *fx*. fracture, *disl*. dislocation, *M* month(s), *Cons*. conservative, *ORIF* open reduction and internal fixation

### Fracture type and concurrent injuries

Based on the abovementioned anatomical classification system, there were four cases of type I, 15 cases of type II, and 10 cases of type III nonunions after a traumatic fracture. Various injuries were incurred at the time of the initial trauma/accident or diagnosis, A comprising a total of 32 concurrent injuries in 18 patients. Nine of these 18 patients had 15 shoulder girdle injuries. There were five coracoid fractures and four acromioclavicular joint dislocations, which were common shoulder girdle injuries. Coracoid fractures were associated only with type II and III fractures [47, 59, 67]. Other concurrent shoulder girdle injuries were scapular neck fracture, scapular body fracture, clavicular fracture, and humeral lesser tuberosity fracture. A suprascapular nerve injury was the only neurovascular injury [47]. The prevalence of shoulder girdle injury for each type of acromion/spine fracture was 25% (1/4) for type I, 33.3% (5/15) for type II, and 30% (3/10) for type III, with no significant difference between fracture types (*p* = 0.95). There were six double disruptions of the superior shoulder suspensory complex (SSSC) [22, 40, 59, 67, 68] and three triple disruptions of the SSSC [47, 67]. Multiple disruptions of the SSSC occurred in 31% (9/29) of the total fractures. Eleven acromion/spine fractures were isolated.

### Period from initial trauma/injury and cause of chronicity

The periods between the initial trauma/accident and final diagnosis of nonunion were clearly described or reasonably estimated for 25 patients. The mean period from initial injury to final diagnosis was 35.2 ± 73.2 months (range 3–360 months). The other four patients were diagnosed with spine/acromion fracture at the time of injury or less than 3 months after injury or symptom onset, but the date of diagnosis of nonunion was unknown. Two patients were treated from the time of injury [69, 70]; one was a 49-year-old male with workers' accident compensation insurance issues whose statements were ambiguous and the author presumed that it was a final stage of a stress fracture [59]; one was a 34-year-old woman with psychiatric problems who was diagnosed with a type III fracture 2 months after open anterior acromioplasty [45].

Among the 25 patients with a known period between injury and diagnosis of nonunion, the most frequent cause of delayed diagnosis of nonunion was conservative treatment for fracture in 11 patients (one type I, seven type II, and three type III fractures), even though their acromion/spine fractures were correctly recognized at the time of the trauma/accident. The next most frequent cause of chronicity was oversight by the physician who had previously examined or treated the patient, which occurred in eight patients (two type I, two type II, and four type III fractures). Among them, a 67-year-old male with a type I fracture that occurred during arthroscopic subacromial decompression underwent immediate open reduction and internal fixation (ORIF) but was diagnosed with a nonunion after 60 months without proper follow-up of the fracture [61]. The other six patients comprised one patient treated conservatively for 3 years for a type II fracture with mild symptoms that had been overlooked for 3 years [71], two patients who did not seek treatment for their fractures [68, 72], and three patients whose reasons for delayed diagnosis of nonunion were unknown [67].

### Reasons for seeking medical advice and treatment methods

Reasons for seeking medical advice were specified for 25 patients who were first diagnosed with nonunion 3 months or more after the injury, excluding two patients with acute traumatic fracture, one patient with an ambiguous statement regarding the injury [59], and one with unknown reason for seeking medical advice [70]. The most common reason for seeking medical advice was unspecified shoulder pain for 20 patients (four type I, nine type II, and seven type III fractures). In most patients, pain occurred during motion and worsened when lifting heavy subjects. Other reasons for seeking medical advice were painful restriction of movement in one patient, restriction of movement in one, and weakness with pain in three. The existence or absence of pain attributable to subacromial impingement was clearly described for eight patients. Subacromial impingement pain was present in six patients (one type I, one type II, and four type III fractures), although two patients with type II fracture had no subacromial impingement pain. The images used for the final diagnosis of nonunion were roentgenography in 14 patients, CT or three-dimensional CT in 14, and unknown in one (Table 2).

**Table 2:** Details of symptoms, treatment methods, and outcomes

Patient' number	Symptoms	Final Tx	Surgical procedure for nonunion	Follow-up period	Outcomes	Remarks
1	Pain	Cons		18 M	Poor	Nonunion, Stress fx.?
2	Painful restriction of ROM	Surg	Bone bridging by iliac crest graft			
3	Complete loss of active movement	Surg	Shoulder arthrodesis			Eden-Hybinette procedure within 3 M after injury
4	Pain	Surg	Plating with cancellous lag screw (compression)	12 M	Excellent	
5	Pain	Surg	Tension band wiring, bone graft	10 M	Excellent	
6	Pain	Cons		18 M	Poor	Nonunion, psychological problems
7	Pain	Surg	Plating, bone graft			
8	Pain	Surg	Plating with screws	16 M	Excellent	
9	Pain	Surg	Tension band wire, bone graft	24 M	Excellent	Final stage of stress fx
10	Pain, SA impingement	Surg	?	24 M	Excellent	Possible os acromiale?
11	Pain over fx., SA impingement	Surg	Cancellous lag screw (compression)	12 M	Excellent	Only compression screw
12	Pain	Surg	Plating + tension band wiring, bone graft	23 M	Excellent	Remove of prominent hard ware (11 M)
13	Pain, Weakness	Surg	Plating, bone graft, decompression of the suprascapular nerve at SG notch	60 M	Excellent	Arthroscopic subacromial decompression for bursal side tear of SSP as a second operation
14	Night pain, Weakness	Surg	Plating, bone graft	5 M	Excellent	
15	Pain	Surg	Plating, bone graft	5 M or more		
16	Pain	Surg	Tension band wiring, bone graft with modified Weaver-Dunn procedure	9 M	Excellent	Traumatic fracture-dislocation of Os acromiale
17	Pain	Surg	Plating with lag screws (compression)	36 M	Excellent	
18	Pain	Surg	Tension band wiring, bone graft	24 M	Poor	Acromial fx. occurred at the first ASD and was fixed with Herbert screw and K-wire. Second ASD was performed 6 M later and CTA head performed 2 years later
19	Pain w/o SA impingement	Surg	Plate, bone graft	24 M	Excellent	
20	Pain, SA impingement	Surg	Plating, bone graft	24 M	Good	SSP suture 1 year after injury (w/o any treatment for spine fracture)
21	Pain, SA impingement	Surg	Plating, bone graft	108 M	Excellent	
22	Pain w/o SA impingement	Cons	Physical therapy			Stable fibrous union?
23		Surg	Plating with interfragmentary compression screw	20 M	Excellent	Plate removal
24		Cons		12 M	Excellent	Nonunion
25	Pain, weakness	Cons	Extracorporeal shock wave therapy			
26		Cons		24 M	Excellent	Plating as first tx., nonunion remained
27	Pain	Surg	Plating with compression screws, bone graft	32 M	Excellent	Required a second procedure using other plates due to failure of the mesh plate
28	Pain	Surg	Plating with compression screws, bone graft	26 M	Poor	Disused osteopenia, heterotrophic ossification around the suprascapular notch and nerve
29	Pain	Surg	Plating with compression screws, bone graft	12 M	Poor	

*SA* subacromial, w/o; without, *Cons*. conservative, *fx*. fracture, *ROM* range of motion, Surg.; surgical, ?; uncertain, *Tx* treatment, SSP supraspinatus, *ASD* arthroscopic subacromial decompression, *CTA* cuff tear arthropathy, *M* month(s)

The nonunion treatment method was clearly described for all 29 patients. Conservative therapy was applied for six patients (five type II fractures and one type III fracture). Among the six patients treated conservatively, one patient with type II fracture was treated using extracorporeal shock wave therapy and achieved successful bone union [73]. Symptomatic nonunion occurred in one patient with a type II fracture with an ambiguous cause and course of injury [59] and one patient with a type III fracture and psychological problems for whom operative treatment was avoided by the physician [45]. The other three patients treated conservatively (a 41-year-old male with plate fixation at the time of injury and two patients treated nonoperatively from the start), had type II fractures and experienced persistent but asymptomatic nonunion with satisfactory function [69, 70, 74].

ORIF was performed in 23 patients (four type I, 10 type II, and nine type III fractures). Although the surgical procedure and fixation method for nonunion varied, the most frequent surgical procedure was plating with bone grafting using iliac bone or removal of local callus tissue in seven patients (two type II and five type III fractures). Plating with inter-fragmental compression screw fixation in the original position was performed in four patients (one type I and three type II fractures). Tension band wiring with bone grafting was performed in four patients (two type I, one type II, and one type III fractures). Plating with inter-fragmental compression screw placement and bone grafting was done in three patients (two type II fractures and one type III fracture). The following procedure was each applied in one patient: plating plus tension band wiring with bone grafting for type II fracture, bridging by iliac bone for type II fracture, and interfragmentary screw fixation for type III fracture. A 59-year-old woman who was injured when she was trapped in a closing tram door underwent the Eden-Hybinette procedure for anterior shoulder dislocation 3 months after injury, but became completely immobile and underwent shoulder arthrodesis 11 months later [22]. One patient underwent an unknown surgical procedure was unknown [75].

Ultimately, some form of bone grafting was performed in 16 cases (73%, 16/22) (for two type I, seven type II, and seven type III fractures). The fixation materials included various plates in 15 cases (for one type I, eight type II, and six type III fractures) and tension band wiring in 5 cases (for two type I fractures, two type II fractures, and one type III fracture). These included one case in which a combination of plating and tension band wiring were used for a proximal type II fracture nonunion [76] (Table 3). There was no significant difference in fixation methods by fracture type (*p* = 0.19). After surgery, one patient required removal of a protruded plate [76], one patient had the plate removed without an explanation of the reason [70], and another required a second operation due to hardware failure [67]. No other intraoperative, postoperative, or late complications have been reported.

**Table 3:** Relationships between fracture types and fixation devices

Fx. type	I	II	III	Total
Plating	1	8	6	15
TBW	2	2*	1	5*
total	3	10*	7	20*

^*^one case was fixed with combination with plating

*Fx*. fracture, *TBW* tension band wiring

### Outcomes

Of 29 patients, 24 patients were followed up for more than 5 months (mean, 24.1 ± 21.3 months). For the other five patients, the follow-up period was unknown for three patients, one patient had only 3.5 months follow-up after shoulder arthrodesis [22], and one had only 3 months follow-up with conservative treatment [74]. Of the 24 patients, one was excluded from the final evaluation because the evaluation using the Oxford shoulder score was questionable and it was unclear whether the presented data were reliable. [48].

Only nine patients were evaluated by widely used evaluation methods such as the Constant score [77], American Shoulder and Elbow Surgeons shoulder score, and Disabilities of the Arm, Shoulder and Hand score. Using the percentage compared with the healthy side [78], we classified these outcomes into four categories: excellent (90–100%), good (80–89%), fair (70–79%), and poor (< 70%). The outcome was also classified into four categories in accordance with the raw Constant score or the raw American Shoulder and Elbow Surgeons shoulder score: excellent (90–100), good (80–89), fair (70–79), and poor (< 70). The Disabilities of the Arm, Shoulder and Hand score was used to define the outcome as excellent (≤ 10), good (≤ 20), fair (≤ 30), or poor (≤ 40). As a result, the outcomes were judged as excellent in 17 patients, good in one, and poor in five. Two of the five patients with poor outcomes were conservatively treated: one patient with a type II fracture without a clear mechanism of injury [59] and one with a type III fracture with psychological problems who developed painful nonunion after the physician chose not to treat the injury surgically [45]. The other three patients whose outcomes were rated as poor were surgically treated. One patient had an intraoperative iatrogenic fracture during arthroscopic subacromial decompression for a massive rotator cuff tear and immediately underwent ORIF, then had a cuff tear arthropathy prosthesis inserted 2 years later without continuous appropriate fracture management, and underwent surgery for type I nonunion 5 years after the injury. [61]. The other two patients were a 34-year-old man who underwent surgery for type III fracture nonunion with disused osteopenia with heterotrophic ossification around the suprascapular notch and nerve at 10 years after gunshot injury, and a 74-year-old woman with a type II fracture sustained more than five months early who had a limited postoperative range of motion and weakness [67].

Of the 19 surgically treated patients with a sufficient follow-up period and clear final results, the outcome was rated as excellent for 79% (15/19) [75% (3/4) for type I, 88.9% (8/9) for type II, and 66.6% for type III fracture nonunion], and as poor for 15.8% (3/19). There was no difference in the excellent rate of each fracture type (*p* = 0.82). Of the six patients treated conservatively, except for the two abovementioned patients with a poor outcome, the following three patients had outcomes rated as excellent despite nonunion: a 35-year-old man with a type II fracture overlooked for 20 months whose symptomatic nonunion converted into the asymptomatic fibrous union after 3 months of physical therapy [74], a 72-year-old man with an acute type II fracture that turned into an asymptomatic nonunion 12 months later [70], and a 41-year-old man who developed asymptomatic nonunion at 24 months after plate fixation for acute type II fracture [69]. The other patient who was treated conservatively and achieved an excellent outcome was a 36-year-old man with a type II fracture that turned into a symptomatic nonunion after 9 months of conservative therapy but achieved bony union using extracorporeal shock wave therapy although there was no description of the follow-up period and functional recovery [73].

## Discussion

There is no uniformity in the naming of fractures lateral to the spinoglenoidal notch that break the continuity of the lateral bony fragment with the main body of the scapula. Furthermore, the previously reported incidences of 8–18% or 9–29% for acromion and/or scapular spine fractures are unreliable because most reporters did not define the extent of the acromion and scapular spine and their boundaries [2, 4, 6–15]. Therefore, the anatomical positions indicated by the terms ‘acromion’ and ‘scapular spine’ should be specified in future scapular fracture statistics and statements. One of the reasons why the distinction between the acromion and the scapular spine has become so confused is presumed to be the schematic diagrams of development presented in the 1930s [34, 79]. In these figures, probably aiming at easier understanding, the acromial ossification centers were roughly illustrated at sites that differed from the positions of the actual ossification centers, and the site indeed extended from the scapular spine and is named the basiacromion [79]. As a result, the term ‘acromion’ is thought to have expanded to include the scapular spine lateral to the spinoglenoidal notch.

No major study has specifically focused on traumatic scapular spine fractures (type III), possibly because such fractures are frequently grouped with fractures of the scapular body or acromion [48]. However, there are many articles about stress fractures of this region secondary to the reverse total shoulder arthroplasty [80]. Our novel classification system of fractures at this region may resolve the terminological confusion and facilitate classification using anatomical landmarks such as the acromial angle and spinoglenoidal notch. Moreover, our classification system considers the extent of the fracture surface and may be useful in the selection of treatment modality and the selection of surgical fixation method and material. From the above-mentioned two characteristics, we believe that our system outperforms any previously reported classification systems. Our new classification system is similar to a fracture classification system after reverse shoulder arthroplasty proposed by Levy et al. [81]. However, although Levy’s classification system uses the attachment sites of each part of the deltoid muscle as indices considering the postoperatively altered mechanical stress in this region, our classification system has no relationship with the attachment sites of the deltoid muscle. Our system is also different to the anatomical classification of Ogawa et al. [26] derived from the actual fracture line, and to Kuhn’s classification that uses displacement and reduction in subacromial space as indices [37].

The ages of the patients with nonunion varied and were distributed over a wide range. In addition, 66%, of our cohort were male, which is agreeance with previous studies that reported a percentage of male patients among all scapular fractures ranging from 64 to 98% [5, 6, 8, 9, 11, 14, 15, 82, 83], and among acromion/spine fractures ranging from 62 to 76% [26, 37, 40]. The cause of injury was traffic accidents and falls or falls from a height in 62% of cases. Two patients had iatrogenic fractures, and it is feared that the number of unreported cases is increasing because the number of shoulder surgeries has increased in recent years. The mechanism of fracture at this site has been reported to be a direct blow [26, 84], the impact caused by falling on the elbow [84], avulsion fracture of the origin of the deltoid muscle [24], and indirect force on the shoulder from the lateral direction [26, 85]. However, among the cases in the present study, the mechanism of injury was clear in only two cases [37, 61].

Fracture types II and III, arising in the anatomical scapular spine from the spinoglenoidal notch to the acromial angle, accounted for 86% (25/29) of fractures. Fewer fractures occurred in the anatomical acromion. In the present study cohort, 38% (11/29) had an isolated fracture of the acromion/spine. The previously reported rates of isolated fractures among patients with traumatic acromion/spine fractures are 24% (6/25) and 16% (6/37) [26, 37]. There is no significant difference for isolated fracture rates of our cohort and reported studies (*p* = 0.13). The abovementioned data suggest that most patients with acromion/spine fractures have associated injuries. Nine patients in our study cohort had multiple disruptions of the SSSC. If a fracture of the acromion/spine with disruption of the SSSC causes unacceptable displacement at either or both sites, surgical management is indicated [38]; thus, these cases require early surgical treatment. However, none of the nine patients with multiple disruptions of the SSSC received early surgical treatment.

Of the 25 patients diagnosed with nonunion 3 months or more after injury, 11 developed nonunion despite conservative treatment from the time of injury. Of these 11 patients, only two had multiple disruptions of the SSSC, which would have required early surgical treatment if the circumstances allowed [40, 47]. As the potential complications of nonoperative management include painful fracture nonunion or increasing fragment displacement [40, 86–88], periodic imaging observations are required during conservative treatment until the bone union is achieved. However, although displacement is the indication for surgery in patients with apophyseal fractures, most authors did not specify the degree of displacement [89]. The fractures were overlooked in eight patients in the present study, which shows how easily fractures in this region are missed by routine roentgenography. Several authors reported that acromion/spine fractures mostly occur in combination with other injuries that are often more severe, and this multitrauma profile of the patient often results in the focus being shifted to another site and can result in a delay in diagnosis [10, 71]. Furthermore, acromion/spine fractures are often undiagnosed at the time of injury and then discovered when the fracture displaces secondary to the deltoid and trapezius muscle tension created during rehabilitation exercises [18]. In fact, the three most common occult fractures in the scapula are fractures of the coracoid process, scapular spine, and glenoid cavity, for which the sensitivity of radiography is much lower than that of multidetector CT [90]. Currently, CT scanning with three-dimensional reconstructions is the most useful imaging modality to detect and define the extent of scapular injury [91]. Therefore, if there is even the slightest suspicion of scapular fracture, it is necessary to perform CT imaging.

Shoulder pain, including subacromial impingement syndrome, was the reason for seeking medical advice in 80% (20/25) of cases in the present review. It was unclear whether the pain emanates from the nonunion or from the subacromial space, as there are few descriptions concerning subacromial impingement (*n* = 8). Since the 1990s, CT and three-dimensional CT have been the most commonly used imaging methods for the final diagnosis of nonunion. CT should be increasingly used in the future not only for detailed observation of nonunion but also as a means of providing useful information for treatment decisions.

ORIF was applied to 23 cases; tension band wiring with bone grafting was mainly used for type I, while plating with bone grafting was used for types II and III nonunion in the present study. Interfragmental compression screws were used together with plating in seven cases. In one of the three surgical cases with a poor final evaluation, the poor result was thought to be caused by an underlying massive rotator cuff tear and resultant cuff tear arthropathy. Serious intra- and postoperative complications occurred in only one patient with hardware failure requiring reoperation. Surgical treatment in this area is considered to have high reliability and safety. Among the conservative treatments, extracorporeal shock wave therapy is considered a useful alternative to surgical treatment or a means of improving the results of surgical treatment [73, 92, 93].

The first internal fixation for a nonunion of the scapular spine was performed by an English surgeon named Robson in 1884 [94]. In 1914, Darrach reported a patient with nonunion of the acromion process at its junction with the spine treated surgically and fixed by silk sutures [95]. Even today, one of the surgical indications for acromion/spine fractures is painful or symptomatic nonunion [40, 96]. Most acromion fractures reportedly heal by the fibrous union without disability [97], and nonunion is not always painful or limiting to shoulder function, especially in elderly or less active patients [70]. However, it is possible that asymptomatic nonunion may become symptomatic in the future, including the three patients in whom nonunion remained but the outcome was evaluated as excellent in the present review [69, 70, 74].

**Fig. 4:**
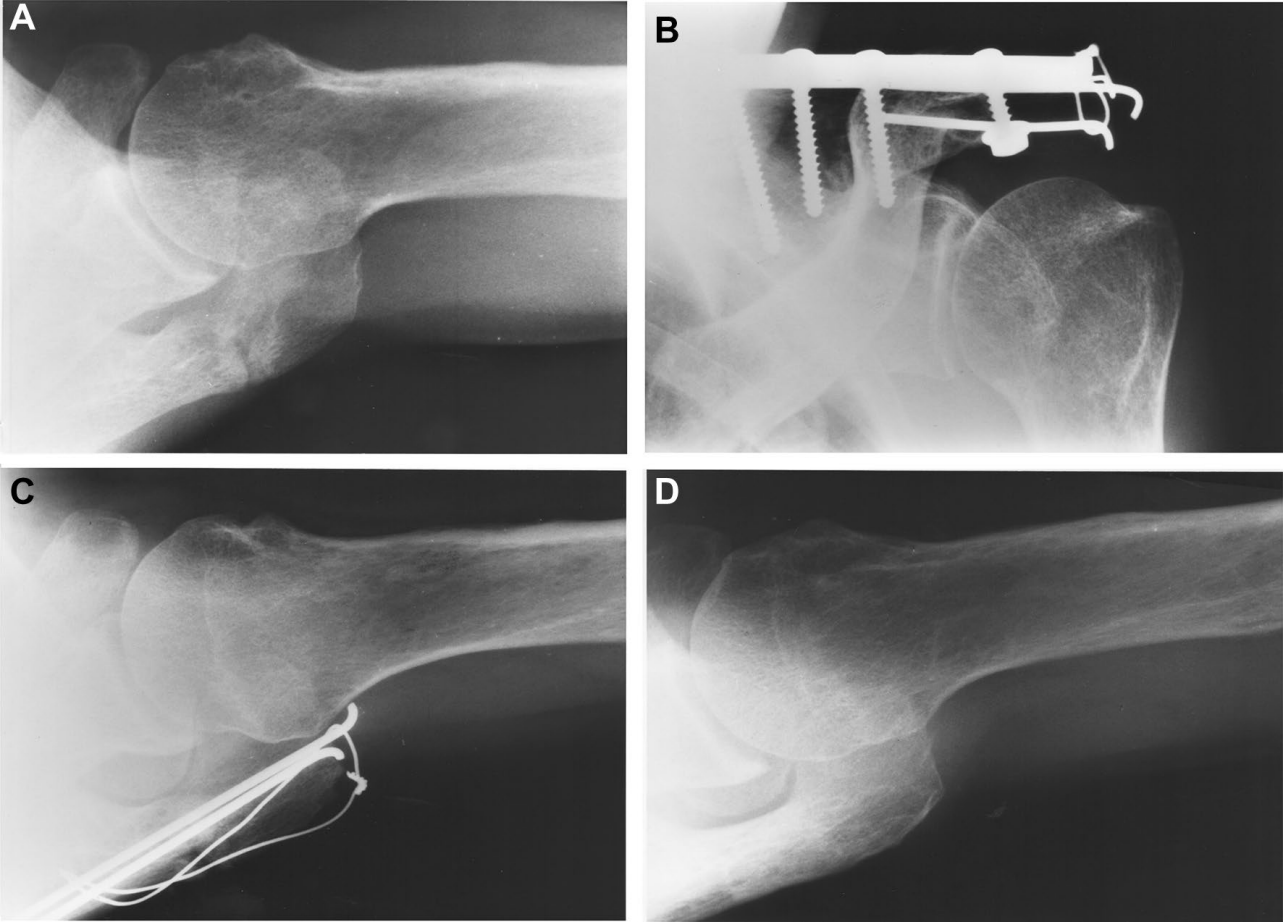
Incorrect selection of the fixation method and material. A 56-year-old man injured his left shoulder by falling heavy lumber. After 5 months of conservative treatment at an alternative medical institution, he underwent tension band wiring at a hospital for type II fracture nonunion (**A**, **B**). However, because pain during movement and at night persisted, the nonunion was repaired at our hospital with a plate, *K*-wires, and bone grafting (**C**) 6 months after the first surgery. Nine months later, he had achieved bone union and a painful fixation material was removed (**D**)

The surgical fixation method should be selected in consideration of the anatomical features and the mechanical stress acting on the fractured part, regardless of whether the injury is a fresh fracture or a nonunion (Fig. 4). Tscherne and Christ reported the use of tension bands to stabilize the lateral acromion fracture and a plate to stabilize the fracture at the transition to the spine [98]; subsequent studies have recommended similar fixation methods [38, 40, 67, 99]. In acromion/spine fractures that form multiple disruptions of the SSSC, the fixation of fractures at this site is important because it reduces and stabilizes fractures at other sites [38, 40, 67, 99].

There are several limitations to this review. The main limitation is that extremely few cases met the inclusion criteria. Therefore, we had to include cases with insufficient information. Most of the included studies are case reports or retrospective case series with small numbers of patients. Additionally, as one case was retrospectively reported by the physical therapist, and details regarding the medical history and functional results were lacking. Additionally, the results of different treatment methods could not be compared due to the variability in outcome evaluation methods. Finally, the variability in the surgical fixation methods made it extremely difficult to perform meaningful comparisons between the outcomes of different fixation methods.

## Conclusion

Isolated acromion/spine fracture nonunion is rare. Fracture types II and III, arising in the anatomical scapular spine, accounted for 86% of the fractures in this study. It is necessary to use periodic imaging observation to achieve early recognition of nonunion and use CT to prevent fracture oversight. Surgical therapy for symptomatic nonunion can produce good stable results, therefore, the nonunion should be treated surgically if the conditions allow. The surgical fixation method and material should be selected after consideration of the anatomical characteristics of the fracture and the stress on the fracture portion.

## Data Availability

The datasets generated during and/or analyzed during the current study are available from the corresponding author on reasonable request.
